# Use of Microalgae Biomass for Fortification of Food Products from Grain

**DOI:** 10.3390/foods10123018

**Published:** 2021-12-05

**Authors:** Julia Bazarnova, Liudmila Nilova, Elena Trukhina, Maya Bernavskaya, Yulia Smyatskaya, Tugba Aktar

**Affiliations:** 1 Department of Institute of Civil Engineering, Peter the Great Sankt-Petersburg Polytechnic University, 194064 Saint Petersburg, Russia; j.bazarnowa2012@yandex.ru (J.B.); nilova_l_p@mail.ru (L.N.); 0946713@mail.ru (E.T.); bernavskaya@mail.ru (M.B.); 2 Department of Food Engineering, Faculty of Engineering, Alanya Alaaddin Keykubat University, 07450 Antalya, Turkey; tugba.aktar@alanya.edu.tr

**Keywords:** *Chlorella* microalgae biomass, pasta, nutrition value, essential lipids, omega 3, omega 6, carotenoids, chlorophyll

## Abstract

This article describes the use of *Chlorella sorokiniana* biomass additives in pasta recipes to enrich the product with biologically active phytonutrients, as well as to achieve the desired color range without the use of synthetic dyes. Samples of dry biomass were obtained by the cultivation of microalgae *C. sorokiniana* (strain), its quality indicators and nutritional value were determined for use as a food additive. A method of using dry biomass of microalgae *C. sorokiniana* as a phytoadditive to replace 5% of flour mixture for effective enrichment of pasta with biologically active phytonutrients was proposed. The choice of the optimal amount of addition of microalgae biomass was proved since it turned out that the replacement of flour should be no more than 5% due to the distinct fish flavor of the final product. The present study was conducted to evaluate the effect of adding dry biomass of *Chlorella* microalgae on total protein, lipid, chlorophyll, and carotenoid content. Substitution of 5% of pasta flour led to an increase in the content of proteins and lipids to 15.7 ± 0.50% and 4.1 ± 0.06%, respectively. Meanwhile, the addition of microalgae *Chlorella* to pasta has helped to increase the content of polyunsaturated fatty acids, chlorophyll, and carotenoids which are necessary for the prevention of foodborne diseases. The aim of this study is to develop pasta recipe with additives of microalgae biomass *C. sorokiniana* and study their quality indicators.

## 1. Introduction

Pasta dishes have been known since the beginning of the first century BC [1]. Traditionally, pasta is made from durum wheat (*Triticum durum*). Although durum wheat contains B vitamins, macro- and microelements, some essential acids (phenylalanine, tryptophan, and isoleucine), the main component is starch, providing a low glycemic index [2,3]. Durum wheat pasta, due to its nutritional properties, low cost, and long shelf life, remains a traditional food product in the diet of many countries. The popularity of pasta is promoted by the variety of dishes that can be prepared from them—as an independent dish [4] or (carbonara pasta, pasta with bolognese sauce, trenette with pesto sauce) or be an ingredient in various dishes, such as lasagna, pizzoccheri ravioli, tortellini, soups.

The nutritional value of pasta can be improved by adding unconventional raw materials rich in dietary fiber [5,6], vitamins, and polyunsaturated fatty acids [7,8,9]. Plant pigments of unconventional raw materials change the sensory properties of pasta (tomato, carrot, spinach), with their simultaneous enrichment, but they cannot enrich PUFA [10,11]. Omega-3 fatty acids, including eicosapentaenoic (EPA) and docosahexaenoic (DHA) acids, are essential since they cannot be synthesized by the human body. Fatty fish such as salmon, mackerel and sardines, fish oil, some nuts, and vegetable oils are the main sources of EPA and DHA in diets, while rapeseed, walnut, soy, and flaxseed oils contain α-linolenic acid (ALA) [12].

A natural source of EPA and DHA is also macroalgae of the *Isochrysidaceae* families (genus *Isochrysis galbana* (Ig) and *Pavlovaceae* (genus *Diacronema vlkianum* (Dv)), which accumulate omega-3 fatty acids, making them an important food raw material obtained in aquaculture [13,14].

Microalgae of the genus *Chlorella* are a promising source of biologically active substances. *Chlorella* biomass is rich in polyunsaturated fatty acids [15], contains protein up to 50%, folic acid, niacin, choline, pantothenic acid, as well as more than 10 types of vitamins, micro- and macroelements, including Ca, K, Fe, Na, Mg, Zn, Cu, P, and Se [16,17,18,19,20]. The chlorophyll content in *Chlorella* reaches 4%, which is 5–10 times more than in the algae *Spirulina* and *Alfalfa* (*Medicago*) [21,22]. Currently *Chlorella* is classified as a food supplement [23,24]. It is widely used in food products for the prevention of iron deficiency, anemia, lowering blood cholesterol levels, etc.

There are some results of clinical trials of *Chlorella* biomass [25] and analysis of its biotechnological potential. The author Muller-Feuga [25] proposed *Chlorella* biomass as an additive for yogurt in order to increase the viability of bacterial probiotics. Emulsions enriched with carotenoids and polyunsaturated fatty acids obtained from the biomass of *Chlorella* microalgae [26,27,28,29], thickened desserts [30], biscuits [31], and pasta [32] have been developed. There is an experience in using *Chlorella* protein hydrolyzate as a food additive [33]. Most often, *Chlorella* biomass is used in the form of a dry powder since in this form it is most bioavailable for human digestive enzymes [34,35].

The aim of this study is to develop pasta recipe with additives of microalgae biomass *C. sorokiniana* and study of their quality indicators.

## 2. Materials and Methods

To obtain the biomass of the *Chlorella* microalgae, a pre-culture of *C. sorokiniana* (*strain 211-8k*) from the collection of algae of the University of Göttingen (Culture Collection of Algae at Göttingen University, international acronym SAG) was used. Biomass cultivation was carried out in a laboratory photobioreactor in the mode of illumination with fluorescent lamps (luminous flux 2500 ± 300 Lx, T (K) 400, daylight, photoperiod—12 h) [34,36]. The cultivation temperature was (23 ± 1) °C; the intensity of aeration of the mixture—1.2–1.8 L/min; mixing mode–periodic (15 min once a day); stirring speed—500 rpm. For the cultivation of microalgae, a culture medium, well-balanced in the content of macro- and microelements, for example MgSO_4_·7H_2_O, KH_2_PO_4_, MnCl_2_·4H_2_O, ZnSO_4_·7H_2_O and other. [37]. The concentration of the cell suspension was carried out by centrifugation at 6000 rpm for 5 min, after which the moist biomass was dehydrated by lyophilization with air cooling Alpha 2–4 Ldplus (pressure 1 MBar, freezing temperature −55 °C). The residual moisture content in dry biomass of microalgae ranged from 3.5 to 4.0%.

While analyzing the chemical composition and biologically active substances in the obtained samples of air-dry biomass of microalgae *C. sorokiniana,* the following characteristics were determined: moisture content by drying [38]; the content of protein, fat and carbohydrates [39]; the fatty acid composition of the allocated lipids [40]; the content of chlorophylls and carotenoids according to the method of the authors Nayek et al. [41]; the content of phenolic compounds by the Folin–Ciocalteu method [42]; microbiological indicators such as *Escherichia coli, Staphylococcus aureus,* and *Salmonella* [43]. When developing pasta recipes with partial substitution of the flour with biomass of microalgae, organoleptic characteristics of pasta after culinary processing was determined [44].

The lipid fraction extraction from *C. sorokiniana* microalgae biomass was carried out on the Büchi E-812 SOX Soxhlet apparatus. For this, 3 g of dry biomass was placed in a cellulose glass (33 mm × 94 mm). A mixture of a solution of ethanol: n-hexane (1:9) was used as an extractant. For 3 g of dry biomass, 100 mL of extractant was used

The composition of higher fatty acids in the sum of lipids obtained from the *Chlorella sorokiniana* microalgae biomass was determined using the gas chromatograph with a flame ionization detector Agilent Technologies Sales & Services GmbH & Co.KG (Waldbronn, Germany), on a BPX70 column (60 m × 0.25 mm × 0.25 μm), SGE Analytical Science, VWR International GmbH; carrier gas was nitrogen.

To prepare the pasta dough, we used commercial durum wheat flour Molino Grassi (manufactured by Molino Grassi S.p.A., Parma, Italy), drinking water, and egg melange. During the development of pasta recipes, we used the replacement of flour with dry biomass of microalgae in an amount of 2.5 to 7.5% by weight of the flour mixture. The preparation of pasta control samples was carried out without the use of microalgae biomass additives. The technology for preparing pasta dough includes the processes of mixing dry components with water, extrusion (extrusion into the required shape), and dehydration (drying under controlled conditions). The dough pieces were molded in the form of a Tagliatelle 7 mm wide and 500 mm long, then dried in an Electrolux oven at 60 °C for 5 h. In dry pasta, the titratable acidity index was determined. The culinary processing of experimental and control samples of pasta was carried out by boiling in water in a ratio of 1:10 (by weight) at a temperature of ~90 °C for 5–7 min until cooked.

The quality of finished pasta was assessed by the increase in the mass and volume of the products during cooking. The preservation of the shape of the products, and the loss of protein substances [45] and chlorophyll [41] was determined.

The determination of the concentration of metal ions in the samples was carried out by anodic stripping voltammetry on the TA-lab analyzer (NPO Tomyanalit LLC, Khimreaktiv, Russia) after mineralization. The concentration of nitrates was determined by photometric method according to the state standard. Statistical processing of the results was carried out. Reagents are manufactured by JSC “Khimreaktiv” Russia.

Samples of pasta were prepared in distilled water until the optimum cooking time, and after drying for 2 min, they were served to experts. Experts rated the products for flavor, appearance, texture, and overall acceptability using a 7-point hedonic scale ranging from 7 (like) to 1 (strongly dislike) for each sensory characteristic [46,47].

## 3. Results

Table 1 shows the general chemical composition of dry biomass of microalgae *C. sorokiniana* obtained by cultivation in the photobioreactor [48,49].

It was determined that dry biomass of microalgae *C. sorokiniana* contains about 48% protein, which is significantly higher than in soy, an alternative source of vegetable protein. Lipid content reached 13% of the biomass sample, which is comparable to or slightly higher than in other aquacultures such as spirulina.

During cultivation, microalgae are able to accumulate metals from the nutrient medium and introduced additives. For further use of biomass in food recipes, it is necessary to establish compliance with the safety indicators of the obtained samples in accordance with the Customs Union Technical Regulations on the safety of food products CU TR 021/2011, according to which the content of toxic elements, nitrates, and microbiological indicators are normalized.

**Figure 1:**
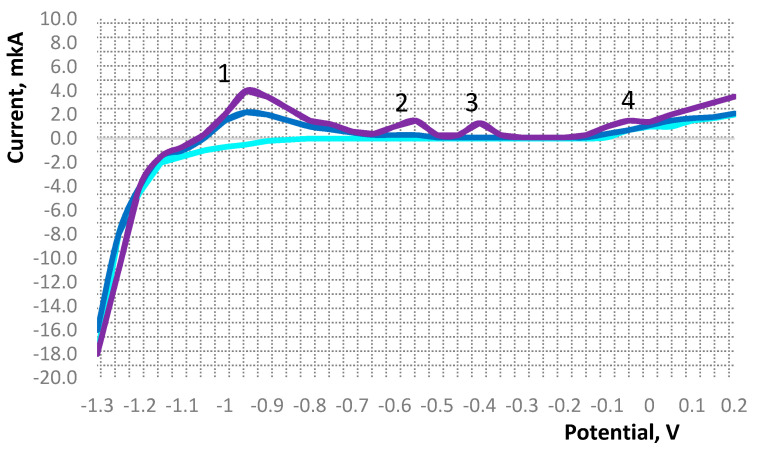
Voltammogram of the test sample. The concentration of metal ions in the sample is proportional to the peak area on the curve: background electrolyte; sample solution; sample with the addition of the target substance with a known concentration. 1—Zn, 2—Cd, 3—Pb, 4—Cu.

Figure 1 shows the results of studying the content of metals in biomass samples by the voltametric method.

The voltammogram shows peaks of current, which serves as an analytical signal, characterizes the nature of the determined element and its concentration in the solution of the electrochemical cell. This figure shows the current peaks for zinc (1), cadmium (2), lead (3), and copper (4). The intensity of the peak signals is the sum of the value of the background current and the electric dissolution current of the determined element concentrate from the surface of the electrode [50].

Table 2 shows the results of studies of the residual content of zinc, copper, and iron in samples of the biomass of microalgae *C. sorokiniana*.

In acceptable amounts, Zn, Cu, and Fe are trace elements, necessary for normal functioning of the human body [51], while lead, cadmium, arsenic, and mercury are toxic elements. Determination of the concentration of toxic elements is necessary to determine the safety of biomass along with the determination of the concentration of nitrates and microbiological indicators.

Table 3 and Table 4 show the results of determining the content of toxic elements and microbiological parameters in the obtained samples of the *C. sorokiniana* biomass.

The content of toxic elements does not exceed the permissible values according to the Customs Union Technical Regulations on safety of food products CU TR 021/2011.

The content of nitrates in biomass is 768 ± 80 mg/kg, which is an acceptable value for biologically active substances.

The content of microbiological indicators complies with the safety standards CU TR 021/2011 and confirms the safety of microalgae biomass.

It is equally important to determine the amino acid and fatty acid composition for understanding the value of food raw materials.

Table 5 shows the composition of a sample of microalgae *C. sorokiniana* dry biomass, obtained by cultivation in a pilot bioreactor [47].

Dry biomass of microalgae *C. sorokiniana* contains all the amino acids necessary for the development of living organisms.

About 80% of the total fatty acid content of *C. sorokiniana* lipids are unsaturated fatty acids. In addition, the biomass is rich in biologically active phytochemicals, along which chlorophylls (22.13 mg/g dry biomass) and carotenoids (6.04 mg/g dry biomass) prevail.

Thus, the inclusion of *C. sorokiniana* biomass additives in pasta recipes will allow enriching products with essential lipids and minor nutritional components, as well as achieving the required pasta color range without the use of synthetic dyes. It was found that the content of phenolic compounds in the of dry biomass samples is about 0.05 mg/g. In previous studies we examined the antioxidant properties of *C. sorokiniana* microalgae and analyzed the total content of phenolic antioxidants in microalgae samples obtained under various light conditions [52]. The content of organic acids in the resulting biomass was about 2.70 mg/g.

**Figure 2:**
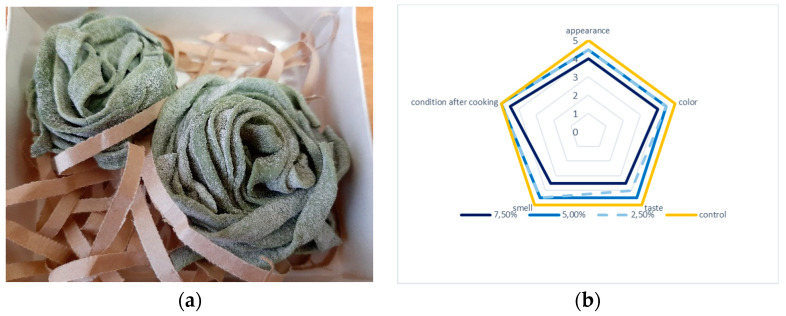
Results of sensory evaluation of finished pasta with replacement of flour with dry biomass of microalgae *C. sorokiniana* (**a**) and tagliatelle samples with 5% biomass additives (**b**).

Figure 2 shows the results of a sensory evaluation of finished pasta with the replacement of a part of the flour in the formulations with dry biomass of *C. sorokiniana* microalgae and pasta samples.

Pasta with microalgae biomass additives had a color from light green to intense green, depending on the amount of additive. It was noted that the samples of pasta with the addition of more than 5% of biomass had a significant fishy flavor. Thus, the addition of microalgae in an amount of more than 5% is impractical.

The results of studies of physicochemical quality indicators of pasta with additives of microalgae *C. sorokiniana* dry biomass are presented in Table 6. 

It was determined that replacing flour with dry biomass of microalgae in the range from 2.5 to 7.5% practically does not affect the moisture loss during drying of pasta, and the loss of dry matter during pasta cooking does not exceed the established values [44].

Protein losses during pasta cooking increase in proportion to the amount of added microalgae additives and vary from 2.6 to 3.4 g per 100 g of finished products.

The introduction of *C. sorokiniana* biomass additives into pasta makes it possible to enrich them with chlorophyll, and its content increases in proportion to the amount of the additive and reaches from 25% to 77% [53]. Valuable composition is possessed not only by *Chlorella*, but also by other types of microalgae [54].

**Figure 3:**
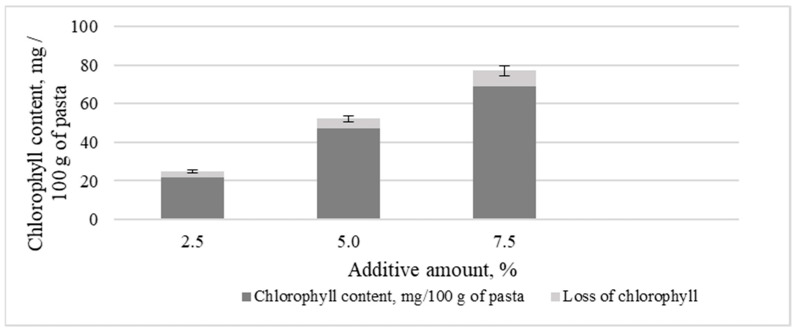
The content of chlorophyll in finished pasta, taking into account its losses during cooking.

Figure 3 shows the chlorophyll content depending on the amount of microalgae biomass additive and chlorophyll loss during pasta cooking.

It was found that the total loss of chlorophyll during pasta cooking is about 10%.

As a result of studies of the shape retention of pasta during cooking, it turned out that the addition of microalgae biomass more than 5% leads to an increase in the digestibility of products.

The presence of gluten in wheat flour promotes the process of swelling and water retention, which contributes to an increase in the volume and weight of products during cooking. The absence of gluten in the composition of microalgae leads to a decrease in water-holding capacity, which entails a slight decrease in the coefficient of increase in the mass of pasta.

Thus, it can be concluded that replacing more than 5% of flour with dry biomass of *C. sorokiniana* microalgae is not advisable.

The nutritional value of pasta is presented in Table 7 [55].

## 4. Discussion

The issue of using microalgae biomass for increasing the nutritional value of pasta has been covered in the literature. There is an experience of using food additives from broccoli [56], amaranth [57], cumini pulp [58], and gac fruit [59] in pasta. The unique composition of *Chlorella* biomass makes it possible to obtain products enriched with the various essential macro- and micronutrients. The addition of 5% dry biomass of *C. sorokiniana* microalgae to flour allowed to increase the protein content by 18% and lipids by 10% in comparison with the control sample; chlorophyll by 34.3%, and carotenoids by 23.6% of the recommended daily intake of these micronutrients according to MR 2.3.1.0253-21.

The authors Lemes et al. [60] note an increase in the content of proteins in pasta up to 14.5% in the case of the addition of *Spirulina platensis* (Cyanobacteria) biomass in the amount of 10%. In the work of the authors Farouk et al. [61], proposed adding the algae *Dunaliella salina* (Chlorophyta) biomass to pasta. The results of studying the composition of pasta showed that the maximum introduction of biomass additive (3% per 100 g of products) allows reaching a protein content of 13.6 ± 0.46%, fats—1.18 ± 0.05%, carbohydrates—83.12 ± 0.75%.

Authors Ozyurt et al. [47], who enriched pasta with the addition of *Arthrospira* (for-merly Spirulina) microalgae biomass in an amount of 10% and obtained a sample with optimal organoleptic properties. The authors Fradique et al. [32] pointed to an improvement in the appearance of pasta with the addition of the biomass of microalgae *Chlorella vulgaris* (Chlorophyta) and *Limnospira maxima* (formerly Spirulina maxima) (Cyano-bacteria), without negative changes in the culinary and textural properties of the finished product. The results obtained on the content of proteins and lipids in the samples of macaroni products with the supplement of 5% microalgae biomass corresponds the literature review.

The carbohydrate content in pasta decreases in proportion to the amount of flour with microalgae biomass substitution. The proposed amount of carbohydrates in flour is about 68%, and in the biomass of *C. sorokiniana* microalgae is about 7% and the calorie content of pasta is 332.8 kcal. Thus, the addition of dry biomass of *C. sorokiniana* microalgae to pasta in the amount of 5% flour mixture does not affect the increase in the calorie content of pasta.

According to research conducted by the authors Klejdus et al. [62], microalgae synthesize isoflavones, flavanones, flavonols, and dihydrohalcones. As a result of the analysis of the total content of phenols and flavonoids in green unicellular algae *Chlorella vulgaris* it has been shown that methanol extracts of *C. vulgaris* contain 220 mg-eq. gallic acid and 131.15 mg-eq. quercitin [63,64]. It is known that the content of phenols in microalgae depends on the composition of the medium and growing conditions. We have previously obtained results indicating that the amount of antioxidant activity of *C. sorokiniana* microalgae biomass correlates with the total content of phenolic compounds and depends on the illumination mode [52].

The disadvantage of using microalgae biomass in the pasta production is the in-stability of unsaturated fatty acids which are subjected to oxidation during the storage. To increase the storage duration, it is proposed to pack products in vacuum bags made of light-proof matte polymer material.

## 5. Conclusions

The dry biomass was obtained by the cultivation of *C. sorokiniana* microalgae (strain 811-K). Chemical composition, physiochemical properties and significant microbiological indicators of biomass were determined. Indicators of the biomass nutritional value were also defined. A method of using dry biomass of *C. sorokiniana* microalgae as a phyto supplement in the recipes of pasta for the effective enrichment with biologically active phytonutrients such as valuable protein, chlorophyl, and carotenoids is proposed.

The introduction of biomass supplement of green microalgae in the recipes of flour products will allow to create natural shades of products which will expand the product range without the use of synthetic additives. In the future, it is planned to conduct research on the development of methods for obtaining essential micronutrients from microalgae biomass, which can be used to replenish their deficiency.

## Figures and Tables

**Table 1:** Specifications and chemical composition of microalgae biomass *C. sorokiniana*.

Components	Content
Appearance	Free-flowing powder
Color	Green
Taste and smell	Fishy, characteristic of algae
Moisture contents, %	~4%
External admixtures	absent
Protein, g/100 g dry biomass	47.82 ± 2.30
Lipids, g/100 g dry biomass	13.32 ± 1.40
Carbohydrates, g/100 g dry biomass	6.90 ± 0.60
Minerals, mg/100 g dry biomass	4.36 ± 0.56

**Table 2:** Residual content of zinc, copper, and iron in biomass samples.

Determined Metal	Content, mg/kg
Zinc	20.20 ± 2.02
Copper	0.720 ± 0.07
Iron	18.3 ± 2.0

**Table 3:** The content of toxic elements in the *C. sorokiniana* biomass.

Indicators	Content, mg/kg
Lead	1.1 ± 0.02
Cadmium	0.66 ± 0.03
Arsenic	0.129 ± 0.01
Mercury	less than 0.002

**Table 4:** Microbiological indicators of the biomass safety of the microalgae *C. sorokiniana*.

Indicators	The Resulting Value
The number of mesophilic, aerobic, and facultative anaerobic microorganisms	2 × 10^3^ CFU/mL
Mold	0 CFU/mL
Yeast	0 CFU/mL
*Escherichia coli* in 1.0 g of product	not detected
*Staphylococcus aureus* in 1.0 g of product	not detected
*Escherichia coli* in 0.1 g of product (coliform)	not detected
*Salmonella* in 25 g of product	not detected

**Table 5:** Indicators of biological value of dry biomass of microalgae *C. sorokiniana*.

Protein, g/100 g		Lipids, g/100 g		Carbohydrates, g/100 g		Minerals, g/100 g	
47.82		13.32		6.90		4.36	
Essential amino acids	mg/g	PUFAs	mg/g	Sugars	mg/g	Phytochemicals	mg/g
Histidine	6.1 ± 0.60	ω_3_	26.6 ± 1.16	Sucrose	204.0 ± 2.00	Chlorophyll	22.13 ± 2.20
Threonine	19.0 ± 2.20	eicosapentanoic	0.53 ± 0.02				
Valine	20.0 ± 1.20	α-linolenic	16.1 ± 0.60	Glucose	133.0 ± 1.20	Phenolic compounds	0.05 ± 0.02
Methionine	1.8 ± 0.20	docosahexaenic	10.0 ± 0.20				
Tryptophan	0.2 ± 0.02	ω_6_	25.7 ± 1.00	Xylose	58.0 ± 0.50	Carotinoids	6.04 ± 0.60
Phenylalanine	18.0 ± 1.00	E-linoleic	3.3 ± 0.05				
Leucine	30.0 ± 2.10	Z-linoleic	14.8 ± 0.50	Fructose	20.0 ± 0.30	Organic acids	2.70 ± 0.40
Lysine	21.0 ± 1.60	octadecatrienoic	7.5 ± 0.10				

**Table 6:** Physical and chemical indicators of the quality of pasta with the addition of microalgae *C. sorokiniana* dry biomass.

Defined Indicators	Percentage of Flour Replacement for Dry Biomass of Microalgae			
	0	2.5	5.0	7.5
Moisture of products, %	8.10 ± 0.40	8.70 ± 0.40	9.70 ± 0.50	10.10 ± 0.50
Acidity ^0^	4.70 ± 0.50	7.10 ± 0.70	7.30 ± 0.70	10.90 ± 0.10
Loss of dry matter during cooking, %	5.00 ± 0.40	5.20 ± 0.40	5.00 ± 0.50	5.00 ± 0.40
Protein loss during cooking, g/100 g finished products	2.40 ± 0.12	2.60 ± 0.13	3.00 ± 0.15	3.40 ± 0.16
Shape retention of finished products, %	100	100	100	98
Volume expansion index	1.10	1.01	1.09	1.10
Product weight increase index	1.62	1.34	1.43	1.44

^0^ According to the Russian standard, acidity is determined by GOST 31964-2012 Macaroni products. Acceptance rules and methods of quality determination acidity is measured in degrees.

**Table 7:** The nutritional value of pasta with the addition of microalgae *Chlorella sorokiniana* dry biomass (5% by weight of the flour mixture).

Proteins, g/100 g	Lipids, g/100 g	Carbohydrates, g/100 g	Calorie Content, kcal/100 g
15.7 ± 0.50	4.1 ± 0.06	57.6 ± 8.64	332.8
Essential fatty acids		Content, mg/100 g	
ω_3_		40.2	
ω_6_		17.8	
Phytochemicals		Content, mg/100 g	
Chlorophyll		52.1	
Carotenoids		3.5	
Organic acids		7.1	

## Data Availability

The data are available from the corresponding author upon reasonable request.

## References

[1] Agnesi E., Krueger J.E., Matsuo R.B., Dick J. (1996). The History of Pasta, in Pasta and Noodle Technology.

[2] Cleary L., Brennan C. (2006). The influence of a (1/3) (1/4)-b-D-glucan rich fraction from barley on the physico-chemical properties and in vitro reducing sugars release of durum wheat pasta. Int. J. Food Sci. Technol..

[3] Tudorica C.M., Kuri V., Brennan C.S. (2002). Nutritional and physicochemical characteristics of dietary fiber enriched pasta. J. Agric. Food Chem..

[4] Feillet P., Dexter J.E., Kruger J.E., Matsuo R.R., Dick J.W. (1996). Quality Requirements of Durum Wheat for Semolina Milling and Pasta Production. Pasta and Noodle Technology.

[5] Brennan C.S., Kuri V., Tudorica C.M. (2003). Inulin-enriched pasta: Effects on textural properties and starch degradation. Food Chem..

[6] Chillo S., Laverse J., Falcone P.M., Protopapa A., Del Nobile M.A. (2008). Influence of the addition of buckwheat flour and durum wheat bran on spaghetti quality. J. Cereal Sci..

[7] Iafelice G., Caboni M.F., Cubadda R., Di Criscio T., Trivisonno M.C., Marconi E. (2008). Development of functional spaghetti enriched with long-chain omega-3 fatty acids. Cereal Chem..

[8] Verardo V., Ferioli F., Riciputi Y., Iafelice G., Marconi E., Caboni M.F. (2009). Evaluation of lipid oxidation in spaghetti pasta enriched with long chain n-3 polyunsaturated fatty acids under different storage conditions. Food Chem..

[9] Bazarnova I.G., Veretnov B.I. (2004). Inhibition of radical oxidation of edible fats by flavonoid Antioxidants. Probl. Nutr..

[10] Di Miceli G., Flagella Z., Marrese P.P., Piro G., Perrotta C., De Bellisb L., Lenucci M.S. (2016). Functional, textural and sensory properties of dry pasta supplemented with lyophilized tomato matrix or with durum wheat bran extracts produced by supercritical carbon dioxide or ultrasound. Food Chem..

[11] Gull A., Prasad K., Kumar P. (2015). Effect of millet flours and carrot pomace on cooking qualities, color and texture of developed pasta. LWT Food Sci. Technol..

[12] Kris-Etherton P.M., Harris W.S.H., Lawrence J.A. (2003). Omega-3 fatty acids and cardiovascular disease: New recom-mendations from the American Heart Association. Arterioscler. Thromb. Vasc. Biol..

[13] Liu C.P., Lin L.P. (2001). Ultrastructural study and lipid formation of Isochrysis sp. CCMP1324. Bot. Bull. Acad. Sin..

[14] Yongmanitchai W., Ward O.P. (1989). Omega-3 fatty acids: Alternative sources of production. Process. Biochem..

[15] Bazarnova Y., Kuznetsova T., Aronova E., Toumi A. (2019). Use of lipids of Chlorella microalgae in poultry meat marinades and sauces recipes. Agron. Res..

[16] Becker E.W., Richmond A. (2004). Microalgae in human and animal nutrition. Handbook of Microalgal Culture.

[17] Liu J., Hu Q. (2013). Handbook of Microalgal Culture: Applied Phycology and Biotechnology.

[18] Chacón-Lee T.L., González-Mariño G.E. (2010). Microalgae for “healthy” foods—Possibilities and challenges. Compr. Rev. Food Sci. Food Saf..

[19] Jachimowicz K., Winiarska-Mieczan A., Baranowska-Wójcik E., Bąkowski M. (2021). Pasta as a Source of Minerals in the Diets of Poles; Effect of Culinary Processing of Pasta on the Content of Minerals. Foods..

[20] Bazarnova Y., Politaeva N., Lyskova N. (2018). Research for the lichen Usnea barbata metabolites Zeitschrift fur Naturforschung—Section C. J. Biosci..

[21] Torres-Tiji Y., Fields F., Mayfield S.P. (2020). Microalgae as a food source of the future. Biotechnol. Adv..

[22] Spoehr H.A. (1951). Chlorella as a source of food. Am. Philos. Soc..

[23] Bishop W.R., Zubeck H.M. (2012). Evaluation of microalgae for use as nutraceuticals and nutritional supplements. J. Nutr. Food Sci..

[24] Becker E.W. (1994). Microalgae Biotechnology and Microbiology.

[25] Muller-Feuga A. (2000). The role of microalgae in aquaculture: Situation and trends. J. Appl. Phycol..

[26] Beheshtipour H., Mortazavian A., Haratian P., Darani K. (2012). Effects of Chlorella vulgaris and Arthrospira platensis addition on viability of probiotic bacteria in yogurt and its biochemical properties. Eur. Food Res. Technol..

[27] Beheshtipour H., Mortazavian A.M., Mohammadi R., Sohrabvandi S., Khosravi-Darani K. (2013). Supplementation of Spirulina platensis and Chlorella vulgaris algae into probiotic fermented milks. Compr. Rev. Food Sci. Food Saf..

[28] Batista A.P., Raymundo S.I., Empis J., Franco J.M. (2006). Colored food emulsions e implications of pigment addition on the rheological behaviour and microstructure. Food Biophys..

[29] Raymundo A., Gouveia L., Batista A.P., Empis J., Sousa I. (2005). Fat mimetic capacity of Chlorella vulgaris biomass in oil-in-water food emulsions stabilized by pea protein. Food Res. Int..

[30] Batista A.P., Gouveia L., Nunes M.C., Franco J.M., Raymundo A., Williams P.A., Phillips G.O. (2008). Microalgae biomass as a novel functional ingredient in mixed gel systems. Gums and Stabilisers for the Food Industry.

[31] Gouveia L., Batista A.P., Raymundo A., Bandarra N.M. (2008). Spirulina maxima and Diacronema vlkianum microalgae in vegetable gelled desserts. Nutr. Food Sci..

[32] Fradique M., Batista A.P., Nunes M.C., Gouveia L., Bandarra N.M., Raymundo A. (2010). Chlorella vulgaris and Spirulina maxima biomass incorporation in pasta products e part I: Preparation and evaluation. J. Sci. Food Agric..

[33] Morris H.J., Almarales A., Carrillo O., Bermúdez R.C. (2008). Utilisation of Chlorella vulgaris cell biomass for the production of enzymatic protein hydrolysates. Bioresour. Technol..

[34] Tarasenko N.A., Tretyakova N.R., Baranova Z.A. Confectionery functional mix for cookies: US Pat. Grew up. Federation- No. 2016143866, 31 July 2017. app. 11/08/2016; publ. 31.07.2017. Bul. No. 22.

[35] Bazarnova Y.G., Kuznetsova T.A., Borgoyakova A.S. (2018). Investigation of the influence of the process of autofloculation of cells of microalgae of microalgae Chlorella sorokiniana in aquaculture on obtaining a complex of pigments. Izvestiya KSTU.

[36] Bazarnova Y.G., Politaeva N.A., Kuznetsova T.A., Tumi A. (2018). Isolation of securities from the biomass of mi-cro-conductor Chlorella sorokiniana. Bull. Technol. Univ..

[37] Bazarnova Y., Kuznetsova T., Boysen H.E. (2018). Methods for concentrating the cell suspension of Chlorella microalgae for obtaining pigment complex. Int. J. Civ. Eng. Technol. (IJCIET).

[38] Guinea G.V., Rojo F.J., Elices M. (2004). Brittle failure of dry spaghetti. Eng. Fail. Anal..

[39] Dawson R.M.C., Elliott D.C., Elliott W.H., Jones K.M. (1986). Data for Biochemical Research, 3rd.

[40] Folch J., Lees M., Stanley G.H.S. (1957). A simple method for the isolation and purification of total lipids from animal tissues. J. Biol. Chem..

[41] Nayek S., Haque C.I., Nishika J., Suprakash R. (2014). Spectrophotometric Analysis of Chlorophylls and Carotenoids from Commonly Grown Fern Species by Using Various Extracting Solvents. Res. J. Chem. Sci..

[42] Singleton V.L., Orthofer R., Lamuela-Raventos R.M. (1999). Analysis of total phenols and other oxidations substractes and antioxidans by means of Folin-Ciocalteu reagent. Methods Enzymol..

[43] Tortorello M. (2003). Indicator Organisms for Safety and Quality—Uses and Methods for Detection: Minireview. J. AOAC Int..

[44] (2000). Approved Laboratory Methods.

[45] Bradford M.M. (1976). A rapid and sensitive method for the quantitation of microgram quantities of protein utilizing the principle of protein–dye binding. Anal. Biochem..

[46] Ozyurt G., Uslu L., Yuvka I., Gökdoğan S., Atci G., Ak B., Isik O. (2015). Evaluation of the Cooking Quality Characteristics of Pasta Enriched with Spirulina Platensis. J. Food Qual..

[47] Otles S. (2011). Methods of Analysis of Food Components and Additives.

[48] Bazarnova Y.G., Kuznetsova T., Trukhina E. (2019). Aquabiotechnology of directed cultivation of microalgae *Chlorella sorokiniana* biomass. IOP Conf. Ser. Earth Environ. Sci..

[49] Petkov G., Garcia G. (2007). Which are the fatty acids of the green alga Chlorella. Biochem. Syst. Ecol..

[50] Politaeva N., Smyatskaya Y., Al Afif R., Pfeifer C., Mukhametova L. (2020). Development of Full-Cycle Utilization of *C. sorokiniana* Microalgae Biomass for Environmental and Food Purposes. Energies.

[51] World Health Organization, Agency, I.A.E. (1996). Nations, Food and Agriculture Organization of the U. Trace Elements in Human Nutrition and Health.

[52] Bazarnova Y., Politaeva N., Kuznetsova T., Toumi A. (2018). Separation of valuable components from *Chlorella sorokiniana* microalgae biomass. Technol. Univ. Bull..

[53] Bazarnova J., Kuznetsova T., Aronova E., Popova L., Pochkaeva E. (2020). A method for obtaining plastid pigments from the biomass of Chlorella microalgae. Agron. Res..

[54] Tolpeznikaite E., Bartkevics V., Ruzauskas M., Pilkaityte R., Viskelis P., Urbonaviciene D., Zavistanaviciute P., Zokaityte E., Ruibys R., Bartkiene E. (2021). Characterization of macro- and microalgae extracts bioactive compounds and micro- and macroelements transition from algae to extract. Foods.

[55] FAO (2010). Fats and fatty acids in human nutrition. Report of an Expert Consultation. Food and Nutrition.

[56] Silva E., Sagis L., Linden E., Scholten E. (2013). Effect of matrix and particle type on rheological, textural and structural properties of broccoli pasta and noodles. J. Food Eng..

[57] Borneo R., Aguirre A. (2008). Chemical composition, cooking quality, and consumer acceptance of pasta made with dried amaranth leaves flour. LWT Food Sci. Technol..

[58] Panghal A., Kaur R., Janghu S., Sharma P., Sharma P., Chhikara N. (2019). Nutritional, phytochemical, functional and sensorial attributes of Syzygium cumini L. pulp incorporated pasta. Food Chem..

[59] Chusak C., Chanbunyawat P., Chumnumduang P., Praew C., Suantawee T., Adisakwattana S. (2020). Effect of gac fruit (*Momordica cochinchinensis*) powder on in vitro starch digestibility, nutritional quality, textural and sensory characteristics of pasta. LWT.

[60] Lemes A.C., Takeuchi K.P., de Carvalho J.C.M., Danesi E.D.G. (2012). Fresh pasta production enriched with Spirulina platensis biomass. Food/Feed Science and Technology. Braz. Arch. Biol. Technol..

[61] El-Baz F.K., Abdo S.M., Hussein A.M.S. (2017). Microalgae Dunaliella salina for use as Food Supplement to Improve Pasta Quality. Int. J. Pharm. Sci. Rev. Res..

[62] Klejdus B., Lojková L., Plaza M., Snyblová M., Stěrbová D. (2010). Hyphenated technique for the extraction and determination of isoflavones in algae: Ultrasound—Assisted supercritical fluid extraction followed by fast chromatography with tandem mass spectrometry. J. Chromatogr..

[63] Zolotareva E.K., Mokrosnop V.M., Stepanov S.S. (2019). Polyphenol compounds of macroscopic and microscopic algae. Algologia.

[64] Jayshree A., Jayashree S., Thangaraju N. (2016). Chlorella vulgaris and Chlamydomonas reinhardtii: Effective antioxidant, antibacterial and anticancer mediators. Indian J. Pharm. Sci..

